# Nutrients as Risk Factors and Treatments for Gestational Diabetes

**DOI:** 10.3390/nu15224716

**Published:** 2023-11-08

**Authors:** Clive J. Petry

**Affiliations:** Institute of Metabolic Science—Metabolic Research Laboratories, Cambridge Biomedical Campus, University of Cambridge, Hills Road, Cambridge CB2 0QQ, UK; cjp1002@cam.ac.uk; Tel.: +44-(0)1223-763200

Gestational diabetes (GDM), traditionally defined as any form of glucose intolerance first detected in pregnancy [1], is one of the most common complications of pregnancy, affecting around 20 million women and their babies per year. The International Diabetes Federation estimated that in 2019 one in every six pregnancies worldwide was affected by it [2]. Poorly-controlled GDM raises the risk of a number of serious short- and long-term complications for both the mother and her baby, including preeclampsia, type 2 diabetes and cardiovascular disease for the mother and stillbirth, large for gestational age, birth trauma and childhood obesity for the baby [3]. The fact that fetal exposure to GDM pregnancies appears to be associated with the long-term risk of GDM (for females) and type 2 diabetes in the offspring has led to some authors proposing that the increasing prevalence is having a major role in driving the diabetes pandemic being experienced worldwide [4].

As maternal overweight and obesity have been described as having a population-attributable fraction of 46% for GDM (using diagnostic criteria appropriate for the first decade of the twenty-first century) [5], at least in the USA, and diet is a major driver of maternal obesity [6], nutrients in the diet and dietary patterns are considered important modifiable risk factors for GDM [7,8,9]. Potential modification of GDM risk does not have to be achieved through altering rates of obesity; however, as consumption of certain micronutrients in pregnancy such as iron and folic acid (which could be consumed as part of the diet, as dietary supplements or both) may increase the risk of GDM [10,11]. Dietary macronutrients have also been linked to increasing GDM risk including the consumption of low fiber, high glycemic index diets [12] and low carbohydrate, high-fat diets [13]. Of late a whole host of dietary strategies have been or are being studied to investigate potential links with alterations in GDM risk, including variations of the Mediterranean-style diet [14,15], high-fiber diets [16], diets with high complex carbohydrate contents [17], plant-based dietary patterns [18], diets high in probiotic yogurt contents [19], and diets with high colorful fruit and vegetable contents [20]. Further, observational dietary studies have reported associations between GDM risk and protein intake in early pregnancy (higher risk with increased early animal protein intakes and lower risk with increased early plant protein intakes [21]). Other dietary approaches currently being tested include a pre-pregnancy lifestyle intervention involving an aerobic exercise regime combined with time-restricted eating of less than ten hours a day, in women who are contemplating pregnancy and have risk factors for GDM [22]. A recently published narrative review about using nutritional approaches to reduce the risk of GDM concluded that despite some dietary factors showing promise for reducing the risk, more studies with large group sizes are required in a variety of different populations before definitive conclusions can be drawn as regards the best dietary modifications to reduce GDM risk [23]. It is possible that more definitive conclusions may require precision medicine approaches, where a recent systematic review and meta-analysis found that combined exercise and dietary approaches to preventing GDM are more effective in women who do not have polycystic ovary syndrome (PCOS) or who have had GDM in previous pregnancies than in women with PCOS or without previous GDM, respectively [24].

Nutrients and diet are linked with GDM in more ways than just potentially altering the risk of disease, however. Diet remains the frontline treatment for GDM [25], even if it needs to be combined with an exercise regime and metformin, glyburide, insulin, or some other pharmacotherapies in some women. So, changes in nutrient intake may reduce pregnancy hyperglycemia. Indeed, diet and lifestyle interventions have been suggested to be some of the top priorities for GDM research according to a group of women with previous experience of pregnancy (including diabetes in pregnancy), support networks, and professionals who answered a survey about perceived top priorities for GDM research [26]. To this end, randomized clinical trials are currently underway, testing effects associated with reduced energy intakes [27] and intermittent- and continuous-low-energy diets in GDM pregnancy [28]. Similar dietary studies have already shown beneficial effects in other forms of diabetes [29,30] or are being tested to that effect [31,32]. Of course, dietary intervention in women with GDM may not just improve maternal glycemia but could also result in improved birth outcomes [33].

One of the current “hot topics” in GDM research and care is when should a formal diagnosis of GDM be made, with fetal changes in GDM-affected pregnancies potentially evident earlier than the 24–28 weeks of pregnancy when it is usually diagnosed [34]. A recently published trial monitoring the effects of treating GDM immediately in women with either GDM or risk factors for hyperglycemia diagnosed before twenty weeks of pregnancy reported that immediate treatment led to a reduction in a composite of adverse neonatal outcomes in comparison to outcomes from pregnancies treated with standard GDM care, even if the effect was modest in size [35]. In terms of how dietary/nutritional modifications could improve this scenario, an optimized healthy diet consumed by women most at risk of GDM, whether from pre-pregnancy or once pregnancy is confirmed, may lead to a reduction in hyperglycemia and a subsequent reduction in adverse neonatal outcomes without needing to be concerned about when a formal diagnosis is made.

Another current “hot topic” in GDM research and care is regarding the optimal treatment regimen for GDM. To that end, a recently published trial investigated whether early treatment with metformin from diagnosis was able to reduce a composite outcome of insulin initiation or incidence of having a fasting plasma glucose concentration of ≥5.1 mmol/L at 32 or 38 weeks of gestation [36]. The trial reported no difference between metformin- and placebo-treated groups in terms of this primary outcome composite variable, although pre-specified secondary outcomes such as lower birth weights and incidence of macrosomia were observed in the metformin-treated pregnancies. Whether or not additional pharmacotherapies are required to treat women with GDM, their glucose tolerance and subsequent to that risk of adverse outcomes [37] would be impacted by the dietary and nutritional intakes. Further work to improve current approaches to the nutritional treatment of GDM is therefore essential.

One further “hot topic” in GDM research and care is GDM-related stigma [38], where it has been suggested that, despite dietary modifications potentially having favorable outcomes on factors such as maternal glycemia and baby’s birth weights [39], women with GDM face major barriers to being able to incorporate nutritional recommendations into their diets [40]. Therefore, in terms of optimizing nutrient intake as a frontline treatment for GDM, for maximal impact, it is likely to require holistic approaches that incorporate both modified nutritional intakes and ways of reducing GDM-related stigma.

For this Special Issue of *Nutrients*, we therefore welcome the submission of manuscripts relating to studies investigating the effects of dietary and nutritional factors that could alter the risk of developing GDM, and those relating to using changes in dietary and nutrient intakes as potential treatments for it.
